# A Novel Strategy to Enhance the pH Stability of Zein Particles through Octenyl Succinic Anhydride-Modified Starch: The Role of Preparation pH

**DOI:** 10.3390/foods13020303

**Published:** 2024-01-18

**Authors:** Linlin Wang, Pengjie Wang, Yi Li, Siyuan Liu, Lida Wu, Weibo Zhang, Chong Chen

**Affiliations:** 1 College of Food Science and Nutritional Engineering, China Agricultural University, Beijing 100083, China; lamlamw@foxmail.com; 2 Key Laboratory of Functional Dairy, Co-Constructed by Ministry of Education and Beijing Government, Department of Nutrition and Health, China Agricultural University, Beijing 100193, China; wpj1019@cau.edu.cn (P.W.); siyuan.liu@cau.edu.cn (S.L.); zhangweibo@cau.edu.cn (W.Z.); 3 Jilin COFCO Biochemistry Co., Ltd., Changchun 130033, China; liyi@cofco.com (Y.L.); wulida@cofco.com (L.W.)

**Keywords:** zein, OSA-modified starch, nanoparticle, stability

## Abstract

Ensuring the stability of zein nanoparticles at different pH levels is crucial for their application as nanocarriers. In this study, octenyl succinic anhydride-modified starch (OSA-modified starch) was employed to enhance the stability of zein nanoparticles against different pH levels by forming complex nanoparticles with OSA-modified starch. The effect of preparation pH on the stability of the zein/OSA-modified starch nanoparticles was investigated. Sedimentation occurred in zein nanoparticles as the pH reached the isoelectric point. However, the stability of zein nanoparticles at various pH levels significantly improved after adding OSA-modified starch to form zein/OSA-modified starch nanoparticles regardless of whether they were prepared under acidic or alkaline pH conditions. Notably, the stability of zein/OSA-modified starch nanoparticles prepared at an acidic pH was higher than that of those prepared at an alkaline pH, thereby highlighting the critical role of the preparation pH for zein/OSA-modified starch in maintaining the stability of zein. The stable zein/OSA-modified starch nanoparticles developed in this study exhibit significant potential for use in delivery systems across various pH environments.

## 1. Introduction

Zein is an alcohol-soluble protein with over 50% hydrophobic amino acids, which is extracted from maize [1]. Due to its hydrophobicity, zein can be used to prepare nanoparticles using the antisolvent precipitation method [2]. During the preparation of zein particles, lipophilic compounds can be co-dissolved in an aqueous ethanol solution and encapsulated within zein particles [1]. Thus, zein has been proven to be a good material for delivering hydrophobic bioactives, nutrients, and drugs to enhance their stability and bioavailability [3]. However, the lower stability of zein in non-acidic pH conditions or around the isoelectric pH limits affects the application of zein nanoparticles [4,5]. Therefore, improving the stability of zein particles against different pH levels is necessary for its application as nanocarriers.

It has been reported that the interactions between protein and polysaccharide significantly influence the properties of nanoparticles, such as conformational stability [6]. To improve the stability of zein nanoparticles against different pH levels, many biopolymers have been used to stabilize zein by forming complex particles [3] such as gum arabic [7], alginate oligosaccharide [2], whey protein isolate [8], and ι-carrageenan [5]. Thus, combining zein with biopolymers has proven to be an effective strategy to prevent zein nanoparticles from aggregation and precipitation over a broad pH range.

Octenyl succinic anhydride-modified starch (OSA-modified starch) is an amphipathic starch derivative created by introducing hydrophobic OSA groups into hydrophilic starch chains through an esterification reaction [9]. OSA-modified starch has been widely used as an emulsifier and encapsulating agent in the food industry due to its amphipathicity [10,11]. OSA-modified starch possesses free carboxyl groups that enable electrostatic interactions with positively charged proteins at pH levels below the protein’s isoelectric point (pI), such as gelatin and casein [12,13]. Furthermore, hydrophobic interactions between proteins and the OSA groups of OSA-modified starch play a crucial role in enhancing the steric stabilization of proteins [14]. Thus, OSA-modified starch is a potential candidate to improve the stability of zein against pH variations.

Recently, OSA-modified starch was used to improve the stability of zein nanoparticles at different NaCl concentrations by forming zein/OSA-modified starch complex nanoparticles using a microfluidic chip [15]. However, the zein/OSA-modified starch complex nanoparticles aggregated as the NaCl concentration increased, even though the addition of OSA-modified starch improved the stability of zein. Another piece of research has focused on the effects of preparation conditions (polar solvents, ultrasonic, and concentrations of OSA-modified starch) on the solubility and thermal stability of zein/OSA-modified starch complex nanoparticles [16]. However, no publications on zein/OSA-modified starch complex nanoparticles focus on their stability against pH variations, even though the instability of zein at the pI has been emphasized. It has been reported that many factors can significantly influence interactions between zein and polysaccharides, such as pH value, ionic strength, and order of polymer addition, which decide the colloidal properties of complex nanoparticles [17]. For example, changing the blending sequences of zein and sophorolipid could produce zein/sophorolipid composite nanoparticles with higher storage stability [18]. Given that the charge of zein varies with the pH, the pH at which zein/OSA-modified starch complexes form may significantly affect interactions between zein and negatively charged OSA-modified starch, thus impacting its stability against different pH levels. Therefore, in this study, OSA-modified starch was used to form complex nanoparticles with zein to enhance the stability of zein nanoparticles across a wide pH range. The effect of zein/OSA-modified starch complexes, which were formed under acidic and basic pH conditions, on zein’s stability against pH variations was further investigated.

## 2. Materials and Methods

### 2.1. Materials

Zein was purchased from Sigma Aldrich (St. Louis, MO, USA). OSA-modified starch (EmulTru12674, derived from non-GM waxy maize with a degree of substitution of 0.024) was obtained from Cargill Investment (China) Co., Ltd. (Shanghai, China). All other chemical reagents used in the study were analytical grade.

### 2.2. Preparation of Zein/OSA-Modified Starch Nanoparticles

Zein powder was dissolved in 75% (*v*/*v*) ethanol/water with continuous stirring for 1 h to a final concentration of 2% (*w*/*v*). OSA-modified starch (4 g) was added in 380 mL of deionized water with continuous stirring for 30 min for complete dissolution. The pH of the zein solution and the OSA-modified starch solution were adjusted to 4.0 or 9.0 with 0.1 mol/L of HCl or NaOH solution, respectively. The zein/OSA-modified starch nanoparticles were prepared using the antisolvent precipitation method [7] with some modifications. A total of 20 mL of 2% (*w*/*v*) zein aqueous ethanol solution was added drop by drop into 380 mL of OSA-modified starch solution with the same pH to fabricate zein/OSA-modified starch nanoparticles. The final ratio of zein to OSA-modified starch was 1:10. The zein solution with the same concentration and pH was selected as the control group.

### 2.3. Characterization of Zein/OSA-Modified Starch Nanoparticles

The pH of zein/OSA-modified starch nanoparticles prepared at pH 4.0 was adjusted from 4.0 to 9.0. Vice versa, the pH of zein/OSA-modified starch nanoparticles prepared at pH 9.0 was adjusted from 9.0 to 4.0. The appearance and particle size of zein nanoparticles were determined to investigate their stability. The appearance of zein/OSA-modified starch nanoparticles solutions with a different pH was recorded to visually evaluate the stability. The zein/OSA-modified starch nanoparticle was diluted to 1 mg/mL with deionized water to measure the zeta potential and size using a Zetasizer Nano-ZS90 (Malvern Instruments, Worcestershire, UK).

### 2.4. Statistical Analysis

All experiments were performed independently three times. The data were expressed as means ± standard deviations. SPSS for windows (version 20) was used to perform the analysis of variance (ANOVA) with the Tukey test. *p* < 0.05 indicated statistical significance.

## 3. Results and Discussion

### 3.1. Appearance of Zein/OSA-Modified Starch Nanoparticles

**Figure 1:**
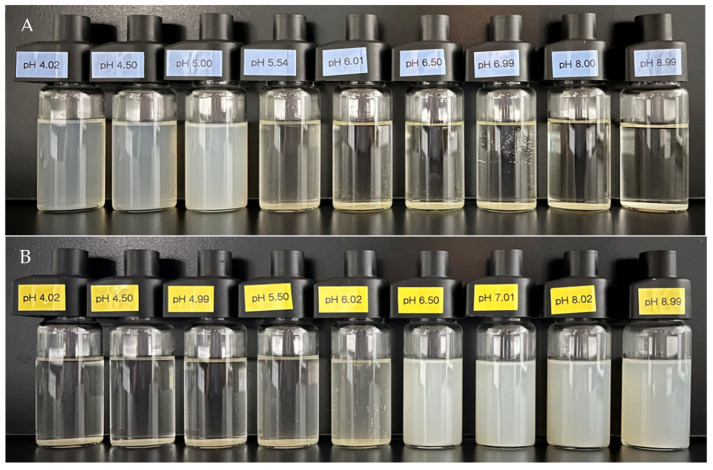
Appearance of zein nanoparticles prepared at pH 4.0 (**A**) and pH 9.0 (**B**) as a function of pH.

The stability of zein nanoparticles against different pH levels is important due to the dramatic pH changes in the human gastrointestinal tract that occur when they are applied as drug nanocarriers. To investigate the pH stability of zein and zein/OSA-modified starch nanoparticles, the appearance of zein nanoparticles and the zein/OSA-modified starch nanoparticle solution prepared at pH 4.0 and pH 9.0 at different pH values was observed and is shown in Figure 1. The zein particles formed at pH 4.0 were homogeneous and stable when the pH was below 5.5. The stable zein particles formed at pH 9.0 were observed when the pH was above 6.0. However, sedimentation occurred in zein nanoparticles when the pH reached the pI of zein (pH 5.5–6.0) regardless of the pH of the zein solutions adjusted from an acidic to an alkaline environment or from an alkaline to an acidic environment. This can be attributed to the aggregation and sedimentation induced by the reduced electrostatic interaction around the pI of zein [1]. Our results confirmed the lower stability of zein around the isoelectric pH, which limited its application as a nanocarrier [4,5].

**Figure 2:**
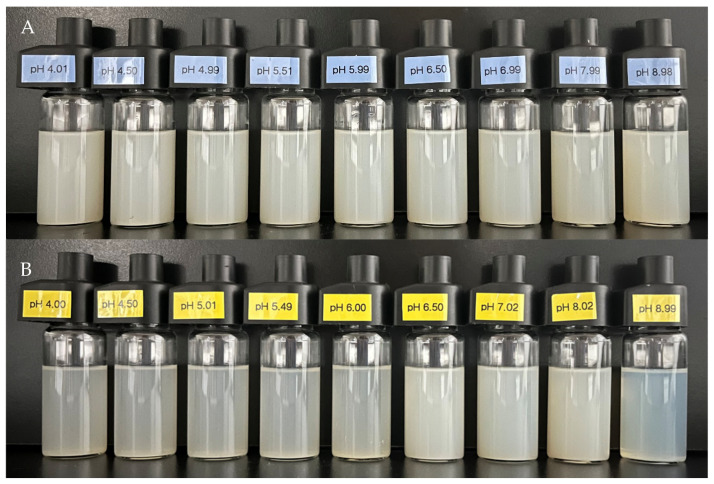
Appearance of zein/OSA-modified starch nanoparticles prepared at pH 4.0 (**A**) and pH 9.0 (**B**) as a function of pH.

However, no sedimentation was observed across all zein/OSA-modified starch nanoparticle solutions within the experiment pH range in Figure 2 regardless of the formation of zein/OSA-modified starch nanoparticles at an acidic or alkaline pH. This result suggested that the addition of OSA-modified starch significantly enhanced the solubility and stability of zein over a wide pH range. The increased electrostatic repulsion or hydrophobic interaction between zein and OSA-modified starch may be the reason for the increased stability [3]. Interestingly, the appearance of zein/OSA-modified starch nanoparticles formed under acidic or alkaline pH conditions differed at the same pH value, which may be attributed to the distinct formation mechanisms of zein/OSA-modified starch nanoparticles.

### 3.2. Particle Size of Zein/OSA-Modified Starch Nanoparticles

**Figure 3:**
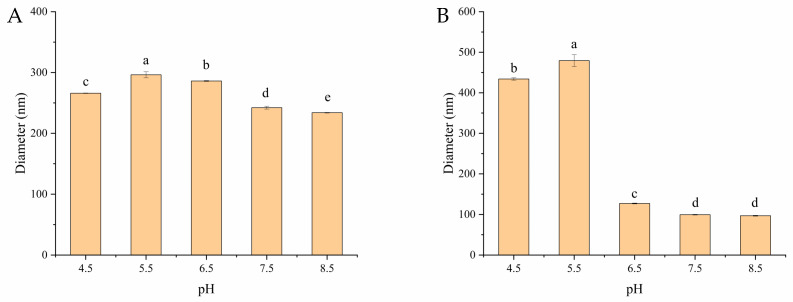
Particle size of zein/OSA-modified starch nanoparticles prepared at pH 4.0 (**A**) and pH 9.0 (**B**) as a function of pH. Different letters above the bars indicate significant differences (*p* < 0.05).

Measuring the changes in the particle size of zein/OSA-modified starch nanoparticles as the pH varied was another method used to investigate the stability of zein as a function of pH. The particle sizes of zein/OSA-modified starch nanoparticles at different pH levels are shown in Figure 3. The particle size of zein/OSA-modified starch nanoparticles prepared at an acidic pH remained 230–290 nm as the pH changed. This result suggested that the zein particles were stable at the measured pH, which is consistent with the appearance results. The reason for the enhanced stability may be the increased electrostatic repulsion after the addition of the negatively charged OSA-modified starch, thereby inhibiting the aggregation of zein nanoparticles [2,12]. However, differing from the appearance results, the particle size of zein/OSA-modified starch nanoparticles prepared in an alkaline pH remained unchanged as the pH decreased until around the pI of zein and then significantly increased as the pH further decreased. This result indicated that the structure of zein/OSA-modified starch nanoparticles changed greatly around the pI of zein, even though no sedimentation occurred. This could be explained through the fact that the formation of zein/OSA-modified starch nanoparticles at pH 9.0 was induced through hydrophobic binding as both polymers were negatively charge. The electrostatic interaction promoted the adsorption and aggregation of zein and OSA-modified starch as the pH crossed the pI of zein [19]. Therefore, the zein/OSA-modified starch nanoparticles prepared at pH 9.0 were not stable enough against different pH levels. These results confirmed that the addition of OSA-modified starch to zein to form zein/OSA-modified starch nanoparticles through electrostatic interaction at an acidic pH significantly improved the stability of zein against pH fluctuations. Thus, the zein/OSA-modified starch complex nanoparticles formed at pH 4.0 showed potential for delivering hydrophobic bioactives, nutrients, and drugs over a wide pH range.

### 3.3. Zeta Potential of Zein/OSA-Modified Starch Nanoparticles

**Figure 4:**
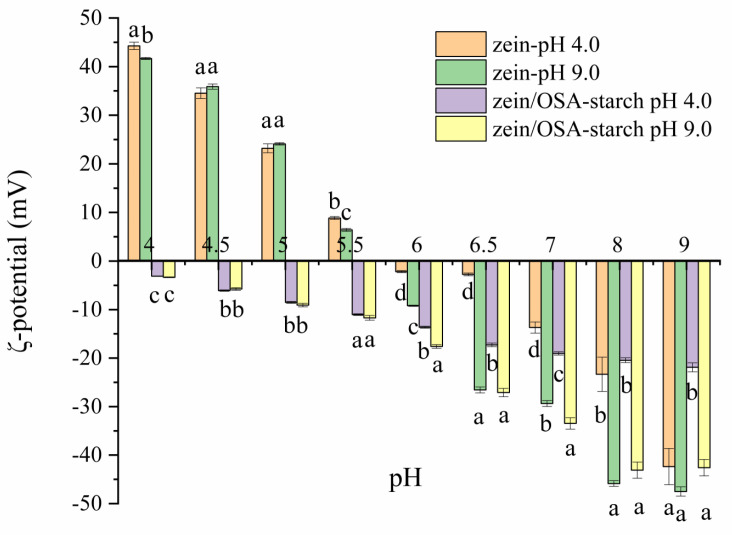
Zeta-potential of zein nanoparticles and zein/OSA-modified starch nanoparticles prepared at different pH levels as a function of the pH. Different letters above the bars at the same pH indicate significant differences (*p* < 0.05).

The zeta potential of zein and zein/OSA-modified starch nanoparticles are shown in Figure 4. As the pH increased from 4.0 to 9.0, the zeta potential of zein nanoparticles changed from 41.67 mV to −47.50 mV, which is consistent with previous reports [20]. The pI was observed to be around pH 5.5–6.0, supporting the findings that the sedimentation of zein nanoparticles occurred within the pH range of 5.5–6.0.

For zein/OSA-modified starch nanoparticles, the zeta potential was negative across the entire pH range, which can be attributed to the charge neutralization of the negatively charged OSA-modified starch [12]. Compared with the zeta potential and stability of zein nanoparticles, no charge conversion occurred in zein/OSA-modified starch nanoparticles, which may be one of the reasons for the stability of zein nanoparticles. The electrostatic repulsion provided through the addition of OSA-modified starch inhibited the aggregation of zein nanoparticles as the pH reached the pI of zein, thereby maintaining the stability of zein particles [2]. However, differences in zeta potential values were observed between zein/OSA-modified starch complex nanoparticles formed at an acidic pH and a basic pH, indicating that the formation mechanism of zein/OSA-modified starch nanoparticles at pH 4.0 and 9.0 may differ. At pH 4.0, positively charged zein particles interacted with negatively charged OSA-modified starch through electrostatic interactions, resulting in charge neutralization and a decrease in zeta potential. As the pH increased from 4.5 to 8.5, the zein absolute potential value continuously increased. These changes in the zeta potential of zein/OSA-modified starch complex nanoparticles prepared at pH 4 with pH were consistent with the results reported by Liu et al. [15]. At pH 9.0, the formation of the zein/OSA-modified starch complex nanoparticles may have been driven through hydrophobic interactions as both zein and OSA-modified starch were negatively charged. Compared with the zein/OSA-modified starch nanoparticles formed through electrostatic interactions, the nanoparticles formed through hydrophobic interactions showed a higher absolute potential value at the same basic pH. This phenomenon can be attributed to the interaction between the hydrophobic groups of OSA-modified starch and zein, with the result being that the free carboxyl groups of OSA-modified starch were exposed to the outside [12,13]. However, as the pH decreased to 5.5, no difference in potential was observed between these two zein/OSA-modified starch nanoparticles. This observation may be attributed to the formation of a new type of zein/OSA-modified starch nanoparticles after the disruption of hydrophobic binding and the occurrence of electrostatic recombination as the pH crossed the pI of zein/OSA-modified starch nanoparticles formed at a basic pH. The changes in the particle size of zein/OSA-modified starch nanoparticles formed at pH 9.0 as a function of the pH supported the explanation regarding changes in zeta potential. The structure of hydrophobic binding in zein/OSA-modified starch nanoparticles significantly changed at the pI. Overall, the pH of the preparation of zein/OSA-modified starch complex nanoparticles played a crucial role in the stability of zein nanoparticles against different pH levels. The zein/OSA-modified starch complex nanoparticles formed at pH 4.0 showed a higher stability across various pH environments.

## 4. Conclusions

In this study, OSA-modified starch was used to stabilize zein against different pH levels by forming zein/OSA-modified starch complex nanoparticles at pH 4.0 and pH 9.0. OSA-modified starch significantly improved the stability and solubility of zein over a wide pH range through increased electrostatic repulsion to inhibit the aggregation of zein nanoparticles. The methods used to form the zein/OSA-modified starch nanoparticles played a crucial role in maintaining the stability of zein as the zein/OSA-modified starch nanoparticles prepared at an acidic pH exhibited superior stability compared with those formed at an alkaline pH when the solution’s pH changed. Disruption of hydrophobic binding of the zein/OSA-modified starch nanoparticles as the pH crossed the pI as well as electrostatic recombination to form new complex particles were the main reasons for the lower stability of zein/OSA-modified starch nanoparticles formed at an alkaline pH. Our research provided a novel and simple method for enhancing the stability of zein nanoparticles by forming complex nanoparticles with OSA-modified starch at an acidic pH, which holds promise for their use in delivering bioactives over a broad pH range as nanocarriers. Future research should focus on the delivery of bioactives through the complex nanoparticles and investigate their bioaccessibility/bioavailability, which are impacted by pH variations in the gastrointestinal environment.

## Data Availability

Data is contained within the article.
